# Validation and Optimization of Host Immunological Bio-Signatures for a Point-of-Care Test for TB Disease

**DOI:** 10.3389/fimmu.2021.607827

**Published:** 2021-02-26

**Authors:** Hygon Mutavhatsindi, Gian D. van der Spuy, Stephanus T. Malherbe, Jayne S. Sutherland, Annemieke Geluk, Harriet Mayanja-Kizza, Amelia C. Crampin, Desta Kassa, Rawleigh Howe, Adane Mihret, Jacob A. Sheehama, Emmanuel Nepolo, Gunar Günther, Hazel M. Dockrell, Paul L. A. M. Corstjens, Kim Stanley, Gerhard Walzl, Novel N. Chegou, the AE-TBC ScreenTB Consortia

**Affiliations:** ^1^ Department of Science and Innovation - National Research Foundation (DSI-NRF) Centre of Excellence for Biomedical Tuberculosis Research, South African Medical Research Council Centre for Tuberculosis Research, Division of Molecular Biology and Human Genetics, Faculty of Medicine and Health Sciences, Stellenbosch University, Cape Town, South Africa; ^2^ TB Research Group, Medical Research Council Gambia at London School of Hygiene and Tropical Medicine (LSHTM), Banjul, Gambia; ^3^ Department of Infectious Diseases, Leiden University Medical Centre, Leiden, Netherlands; ^4^ Department of Internal Medicine and Immunology, School of Medicine, Makerere University, Kampala, Uganda; ^5^ Karonga Prevention Study, London School of Hygiene and Tropical Medicine, Karonga, Malawi; ^6^ Infectious and Non-Infectious Diseases Research Directorate, Ethiopian Health and Nutrition Research Institute, Addis Ababa, Ethiopia; ^7^ Department of Immunology, Armauer Hansen Research Institute, Addis Ababa, Ethiopia; ^8^ Department of Biochemistry & Microbiology, School of Medicine, University of Namibia, Windhoek, Namibia; ^9^ Department of Infection Biology, Faculty of Infectious and Tropical Diseases, London School of Hygiene and Tropical Medicine, London, United Kingdom; ^10^ Department of Molecular Cell Biology, Leiden University Medical Centre, Leiden, Netherlands

**Keywords:** bio-signature, diagnostic, validation, blood, biomarkers, point of care, *M. tuberculosis (M. tb)*

## Abstract

The development of a non-sputum-based, point-of-care diagnostic test for tuberculosis (TB) is a priority in the global effort to combat this disease, particularly in resource-constrained settings. Previous studies have identified host biomarker signatures which showed potential, but there is a need to validate and refine these for development as a test. We recruited 1,403 adults presenting with symptoms suggestive of pulmonary TB at primary healthcare clinics in six countries from West, East and Southern Africa. Of the study cohort, 326 were diagnosed with TB and 787 with other respiratory diseases, from whom we randomly selected 1005 participants. Using Luminex^®^ technology, we measured the levels of 20 host biomarkers in serum samples which we used to evaluate the diagnostic accuracy of previously identified and novel bio-signatures. Our previously identified seven-marker bio-signature did not perform well (sensitivity: 89%, specificity: 60%). We also identified an optimal, two-marker bio-signature with a sensitivity of 94% and specificity of 69% in patients with no history of previous TB. This signature performed slightly better than C-reactive protein (CRP) alone. The cut-off value for a positive diagnosis differed for human immuno-deficiency virus (HIV)-positive and -negative individuals. Notably, we also found that no signature was able to diagnose TB adequately in patients with a prior history of the disease. We have identified a two-marker, pan-African bio-signature which is more robust than CRP alone and meets the World Health Organization (WHO) target product profile requirements for a triage test in both HIV-negative and HIV-positive individuals. This signature could be incorporated into a point-of-care device, greatly reducing the necessity for expensive confirmatory diagnostics and potentially reducing the number of cases currently lost to follow-up. It might also potentially be useful with individuals unable to provide sputum or with paucibacillary disease. We suggest that the performance of TB diagnostic signatures can be improved by incorporating the HIV-status of the patient. We further suggest that only patients who have never had TB be subjected to a triage test and that those with a history of previous TB be evaluated using more direct diagnostic techniques.

## Introduction

Tuberculosis (TB) remains a major global health burden with the World Health Organization (WHO) reporting 10.4 million new TB cases and 1.6 million TB-related deaths worldwide in 2018. TB is also the leading cause of death for people infected with the human immunodeficiency viruses (HIV) (1). The lack of rapid, accurate, point-of-care diagnostic tests poses a serious challenge to control efforts (2). The Ziehl Nielsen sputum smear test is often the only affordable diagnostic tool available in resource constrained environments, even though its limitations, notably its low sensitivity, are well publicized (3, 4). *Mycobacterium tuberculosis (M.tb)* culture, the reference test, is not widely available in these settings. It also has several drawbacks which include a long turnaround time, high costs, and significant rate of contamination (3). The GeneXpert^®^ MTB/RIF sputum test (Cepheid, USA), one of the major advances in TB diagnosis, produces results within 2 h and is coupled with the detection of rifampicin resistance. The GeneXpert^®^ test is widely available in developed countries but limitations, including relatively high operating costs and infrastructural requirements hinder its use in resource-constrained settings (5, 6). It also has significantly reduced sensitivity in paucibacillary disease although this has been somewhat remedied by the newer Xpert Ultra^®^ (7). A common and very important limitation of the above-mentioned diagnostic tests is that they are all sputum-based which renders them unsuitable for use in individuals who have difficulty in providing good quality sputum. This is particularly true of children, who typically develop paucibacillary disease (8), and also of individuals with extra-pulmonary TB or who are HIV-positive. There is, therefore, an urgent need for alternative diagnostic tests that are suitable for use in all patient types, especially in resource-poor settings (9).

Transcriptome-based diagnostic markers have attracted a good deal of interest as an alternative to current tests. They are particularly attractive as detection technologies are well established and require minimal adaptation, irrespective of the target markers. While this approach shows some promise and has the advantage of being relatively easy to implement, as a result of small sample numbers or inappropriate controls, most studies to date have failed to yield a performance which meets the requirements of the WHO for a viable diagnostic (10).

Immunodiagnostics have received considerable attention as an alternative for the detection of TB disease in recent years (11, 12). They are particularly promising as they could be developed into point-of-care tests, which would be easily accessible to resource limited settings. Such tests would also be useful in cases where a sputum-based diagnosis (GeneXpert^®^, smear microscopy or culture) is difficult or not available (13). The emergence of interferon gamma release assays (IGRAs) was a prominent advancement in the development of immunodiagnostic tools for *M.tb*. Commercially available IGRAs rely on the reaction of the immune system to antigens encoded within the region of difference 1 (RD1) of the *M.tb* genome, namely early secreted antigenic target 6 (ESAT-6) and culture filtrate protein 10 (CFP-10) (14). These assays are useful in diagnosis of infection with *M.tb*, however, they are of limited value in high TB-endemic areas as they cannot discriminate between active TB and latent *M.tb* infection. Attempts have been made to identify antigens that may be useful in the diagnosis of active TB (15–17). However, the requirement for overnight culture precludes the use of these assays as point-of-care tests.

Host immunological markers detected in *ex vivo* samples have shown potential for the diagnosis of TB disease (13, 18–21). These markers may be incorporated into a field-friendly, point-of-care test based on finger-prick blood and lateral flow technology. One such test, currently under development, relies on a seven-marker host bio-signature identified in serum (20). However, tests based on large signatures such as this are expensive, complex to design and manufacture and rely on the continued production of numerous components by suppliers. It would, therefore, be of benefit to devise a small, reliable bio-signature implementable in a test device that would be cost-effective and relatively simple to produce. One such signature that has been previous identified in a high-TB incidence setting in HIV-positive participants is C-reactive protein (CRP) (22). It appears to be one of the more promising markers and consequently worth validating in this study in a broader context.

An important characteristic of TB, which complicates its diagnosis, is its frequent association with HIV infection (23). Any diagnostic test that is to be useful should be capable of detecting TB in both HIV-positive and -negative patients. Immunological responses of patients to *M.tb* may differ, dependent on their genetic profiles as well as the bacterial strain with which they are infected (24). It is, therefore, important that a diagnostic test be sufficiently robust as to yield valid results irrespective of host-genetic background and prevalent *M.tb* strains.

This study investigated the potential of several previously identified, serum protein host markers to detect pulmonary TB in patients presenting at primary healthcare clinics, in seven sites across six African countries, with symptoms indicating possible TB (20). We further aimed to investigate the diagnostic potential of modified bio-signatures identified by us and combinations of other markers from the literature (20, 21). The study comprised a large cohort of HIV-negative and HIV-positive participants from different regions of the African continent in order to ensure that any bio-signature identified would be widely applicable in a point-of-care test for TB disease.

## Methods

### Study Participants

**Figure 1:**
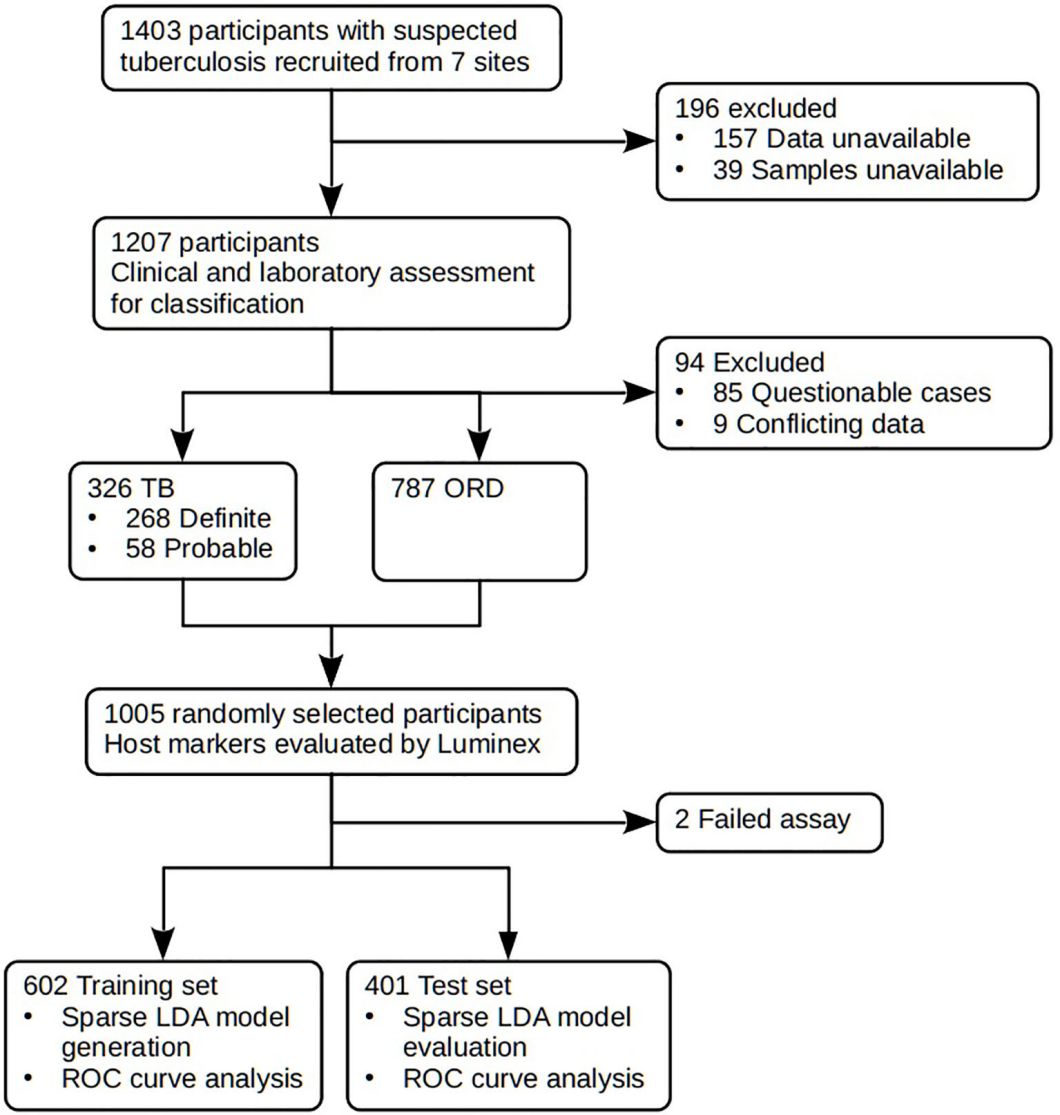
STARD diagram showing the study design and classification of study participants. TB, pulmonary TB; ORD, individuals presenting with symptoms and investigated for pulmonary TB but in whom TB disease was ruled out; ROC, receiver operator characteristic. STARD, Standards for Reporting of Diagnostic Accuracy.

We prospectively recruited 1,403 adults (18 years or older) (**Figure 1**), presenting with symptoms suggestive of pulmonary TB disease at primary healthcare clinics at seven field sites in six African countries as previously described (20). Both HIV-positive and -negative participants were included. Participants in this study were recruited as part of the EDCTP-funded African-European Tuberculosis Consortium (AE-TBC) which included Stellenbosch University (SUN), South Africa; Makerere University, Uganda (UCRC); Medical Research Council Unit, The Gambia at the London School of Hygiene and Tropical Medicine (MRCG); Karonga Prevention Study (KPS), Malawi; University of Namibia (UNAM), Namibia; Ethiopian Health and Nutrition Research Institute (EHNRI), Ethiopia and The Armauer Hansen Research Institute (AHRI), Ethiopia.

Criteria for inclusion in the study were a cough persisting for more than 2 weeks together with any one of the following: fever, recent weight-loss, night-sweats, hemoptysis, chest-pain, or anorexia. Participants were included in the study if they were 18 years or older, willing to give written informed consent, including for HIV testing using a rapid test (Abbott, Germany), and sample storage. Exclusion criteria for the study included severe anemia (hemoglobin <10g/l), pregnancy, other known chronic diseases such as diabetes mellitus, current anti-TB treatment, anti-TB treatment in the last 3 months, use of quinolone or aminoglycoside antibiotics in the past 2 months, or residency in the study area for less than 3 months. Study participants were recruited between November 2010 and November 2012.

Approval for the study was granted by the Human Research Ethics Committee of Stellenbosch University (N10/08/274) as well as the ethics committees of the respective partner institutions.

### Classification of Study Participants

Prior to the commencement of recruitment, harmonized case definitions were established to be used for the classification of study participants at all study sites. Using a combination of clinical, radiological, and laboratory findings, participants were classified as either definite TB cases, probable TB cases, or other respiratory disease (ORD) as previously described (20). The ORD cases were participants diagnosed as having a range of other respiratory conditions including upper and lower respiratory tract bacterial or viral infections (although no attempt was made to identify organisms by culture), and acute exacerbations of chronic obstructive pulmonary disease or asthma. In assessing the diagnostic accuracy of the markers investigated in the present study, all the definite and probable TB cases were classified as “TB,” and then compared to the ORD cases (**Figure 1**). Participants who could not be diagnosed with an acceptable degree of certainty due to insufficient or contradictory clinical evidence (**Table 2**) were excluded from the analysis.

### Reading of Chest X-Rays

Each chest X-ray was reviewed by research medical officers at each site, who decided whether the quality was adequate for classification and then classified it as either normal, abnormal and suggestive of active TB, or abnormal and not suggestive of active TB. Repeat X-rays were compared to baseline X-rays and classified as wither resolved, improved, unchanged, or deteriorated. Lesion types were also documented for abnormal X-rays. The central study clinician reviewed X-rays of all participants whose main study classification (definite TB, probable Tb, No TB) was dependent on the chest X-ray findings. Cases in which the central study clinician’s opinion differed from that of the research medical officer were discussed with the study PI.

### Sample Collection and Microbiological Diagnostic Tests

Harmonized protocols were used for collection and processing of samples across all study sites. Briefly, blood samples were collected at first contact with the patient in 4ml plain BD Vacutainer serum tubes (BD Biosciences) and transported within 3 h at ambient temperature to the laboratory. Tubes were then centrifuged at 2,500 rpm for 10 min, after which serum was harvested, aliquoted into bar-coded cryotubes, and frozen at –80°C until use. Sputum samples were collected from all participants and cultured using either the *Mycobacteria* growth indicator tube (MGIT) method (BD Biosciences) or Lowenstein–Jensen media, depending on facilities available at the study site. Specimens demonstrating growth of micro-organisms were examined for acid-fast bacilli using the Ziehl–Neelsen method followed by either Capilia TB testing (TAUNS, Numazu, Japan) or polymerase chain reaction (PCR) to confirm the isolation of organisms of the *M.tb* complex before being designated as positive cultures.

### Luminex^®^ Multiplex Immunoassay

Using Luminex^®^ technology, we measured the levels of 20 host biomarkers using antibodies supplied by Merck Millipore, Billerica, Massachusetts, USA and R&D Systems, Minneapolis, Minnesota, USA (**Table 1**). All samples were evaluated undiluted or diluted according to the manufacturers recommendations. Samples were randomized to assay plates with the experimenter blinded to sample data. All assays were performed and read in a central laboratory (SUN) on the Bio-Plex platform (Bio-Rad), with the Bio-Plex Manager Software (ver. 6·1) used for bead acquisition and analysis.

**Table 1:** Host markers evaluated in this study nomenclature.

Abbreviation	Full Name
*Merck Millipore, Billerica, Massachusetts, USA*	
CRP	C-reactive protein
SAA	Serum amyloid A
SAP	Serum amyloid P component
FIBR	Fibrinogen
NCAM	Neural cell adhesion molecule
ApoA1	Apolipoprotein A1
Apo-CIII	Apolipoprotein C-III
CFH	Complement factor H
*R&D Systems, Minneapolis, Minnesota, USA*	
TGF-α	Transforming growth factor alpha
IFN-γ	Interferon gamma
IP-10	IFN-γ-inducible protein
TNF-α	Tumour necrosis factor alpha
Serpin C	Serpin C
Ferritin	Ferritin
CCL14/HCC-1	Chemokine (C-C motif) ligand 14
CCL1/I-309	Chemokine (C-C motif) ligand 1
MIG/CXCL9	Monokine induced by gamma interferon
VEGF-A	Vascular endothelial growth factor A
BNDF	Brain-derived neurotrophic factor
GDF15	Growth/differentiation factor 15

### Data Management and Statistical Analysis

All participant and laboratory data were captured using a central REDCap database hosted at SUN (25). Participant and sample management was done using a multi-site study management REDCap plugin application developed at SUN.

All statistical analysis was done using R (ver. 3·6·1) [R Core Team (2019). R: A language and environment for statistical computing. R Foundation for Statistical Computing, Vienna, Austria. URL https://www.R-project.org/] working in the RStudio (ver. 1·2·5019) environment (RStudio Team (2019), RStudio: Integrated Development for R. RStudio, Inc., Boston, MA URL http://www.rstudio.com/) running on Kubuntu Linux 19·04. Unless otherwise stated, graphs were produced using the ggplot2 package (ver. 3·2·1). Parallel processing was facilitated by the doParallel package.

Missing values for SAA fluorescence data were imputed for 42 records using the missForest package using sex, age, HIV status, TB status, and all analytes as predictors.

Due to the presence of a few extreme fluorescence values for some of the analytes, the fluorescence data were subjected to winsorization whereby values greater than 10 median absolute deviations from the median were proportionally shrunk toward the median absolute deviation as calculated with the exclusion of the outliers.

Data were randomly divided into a training set (60%) and a test set (40%). Representivity of each study site in the training set ranged from 55 to 66% of the site’s data.

Sparse Linear Discriminant Analysis (sparseLDA) models were trained using the Caret package (ver. 6.0-84) with predictor variables normalized using the YeoJohnson transformation, centered and scaled. Smote resampling was used to balance the proportions of TB and non-TB cases. Models were optimized for maximum sensitivity at a minimum specificity of 0.7 using a customized version of Caret’s twoClassSummary function.

A model based on a previously published combination of 7 markers was trained using 32-times repeated, 10-fold cross-validation to select the optimal number of terms and the value for the regularization parameter, lambda. The model allowed interactions between the predictors.

An optimal model was developed by first selecting a set of markers by training a model using 32-times repeated, 10-fold cross-validation without interactions. These markers were then used to generate a new model, which allowed interactions between the predictors, using cross-validation to select the optimal number of terms and value for the regularization term, lambda, which reduces the risk of overfitting.

A model, weighted to compensate for the lower prevalence of HIV-positive cases in the dataset, was generated similarly, but with the addition of a weights vector to the caret::train function. HIV-positive cases were assigned a weight inversely proportional to the number of HIV-positive cases and likewise for HIV-negative cases.

Receiver-operator characteristic (ROC) curves were produced using the pROC package. The 95% confidence intervals for sensitivity were generated by bootstrap resampling (R=10,000). Optimal cut-off values were selected as the highest sensitivity such that specificity remained above 70% where possible. Area under the curve (AUC) values were compared using the bootstrap method.

## Results

### Clinical and Demographic Characteristics of Study Participants

In the current study we recruited a total of 1,403 participants (**Figure 1**). Of these, 196 participants were excluded due to the unavailability of data or samples. Using pre-established case definitions (**Table 2**), 268 (22.2%) of the remaining 1,207 study participants were classified as definite pulmonary TB cases and 58 (4.8%) as probable TB cases, together representing the active TB group (326 participants; 27.0%). 787 (65.2%) were classified as ORD cases. Ninety-four (7·8%) participants could not be reliably classified due to incomplete data or loss to follow-up and were excluded from the study. 1,005 of the remaining participants were randomly selected for further analysis. Demographic and clinical details of the participants are given in **Table 3**.

**Table 2:** Classification definitions used to identify study participants groups.

Classification	Definition
Definite TB	Sputum culture-positive for MTB.OR2 positive smears and symptoms responding to TB treatment.OR1 positive smear plus CXR suggestive of PTB
Probable TB	1 positive smear and symptoms responding to TB treatment.ORCXR evidence and symptoms responding to TB treatment.
ORD	Negative cultures, negative smears, negative CXR and treatmentnever initiated by healthcare providers

CXR, chest X-ray; MTB, Mycobacterium tuberculosis; ORD, other respiratory disease; TB, pulmonary TB. (Reproduced from Chegou et al., Thorax 2016) (20).

**Table 3:** Clinical and demographic characteristics of analyzed study participants from the seven study sites.

Study site	AHRI	EHNRI	KPS	MRCG	SUN	UCRC	UNAM	Total
**Participants**	149	185	109	188	156	170	46	1003
**Age in years, mean ± SD**	33·4 ± 11·6	35·1 ± 14·7	39·7 ± 13·8	35·4 ± 12·6	37·5 ± 11·5	32·6 ± 9·9	35·6 ± 11·0	35·4 ± 12·5
**Males, n (%)**	75 (50·3)	76 (41·1)	56 (51·3)	109 (58·0)	64 (41·0)	87 (51·2)	27 (58·7)	494 (49·3)
**Previous TB, n (%)**	20 (13·4)	19 (10·2)	9 (8·3)	14 (7·4)	65 (41·7)	4 (2·4)	11 (23·9)	142 (14·2)
**HIV pos TB pos, n (%)**	6 (10·7)	13 (24·1)	12 (60·0)	8 (15·1)	4 (16·0)	7 (11·3)	15 (58·3)	71 (25·5)
**HIV pos TB neg, n (%)**	5 (4·1)	16 (12·0)	49 (55·7)	9 (6·7)	24 (18·3)	21 (19·4)	5 (50·0)	129 (17·8)
**Definite TB, n (%)**	28 (18·8)	53 (28·6)	17 (15·6)	44 (23·4)	21 (13·5)	58 (34·1)	31 (67·4)	252 (25·1)
**Probable TB, n (%)**	0 (0)	0 (0)	3 (2·8)	9 (4·8)	4 (2·6)	4 (2·4)	5 (10·9)	25 (2·5)
**Total TB, n (%)**	28 (18·8)	53 (28·6)	20 (18·3)	53 (28·2)	25 (16·0)	62 (36·5)	36 (78·3)	277 (27·6)
**ORD, n (%)**	121 (81·2)	132 (71·4)	89 (81·7)	135 (71·8)	131 (84·0)	108 (63·5)	10 (21·7)	726 (72·4)
**ORD QFTpos/tested, (%)**	ND	ND	29/86 (33·7)	40/129 (31·0)	86/127 (67·7)	59/104 (56·7)	7/10 (70·0)	224/456 (49·1)

TotalTB, sum of definite and probable TB cases; ORD QFT, quantiferon results for ORD participants.

### Performance of Individual Serum Biomarkers in the Diagnosis of TB Disease

**Figure 2:**
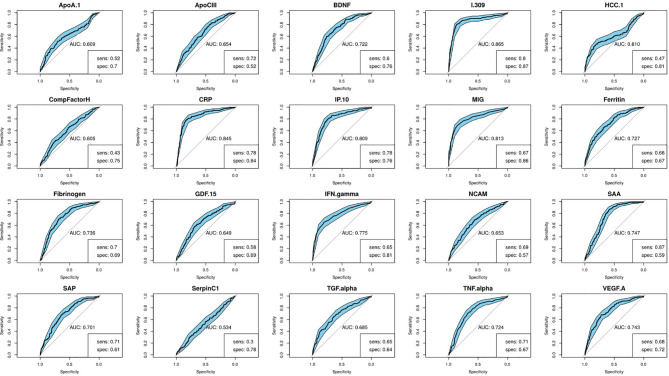
Receiver-operator characteristic (ROC) curves of the diagnostic performance of the individual biomarkers in the training set. Sensitivity and specificity are given for the maximized Youden’s J statistic cut-off point.

**Figure 3:**
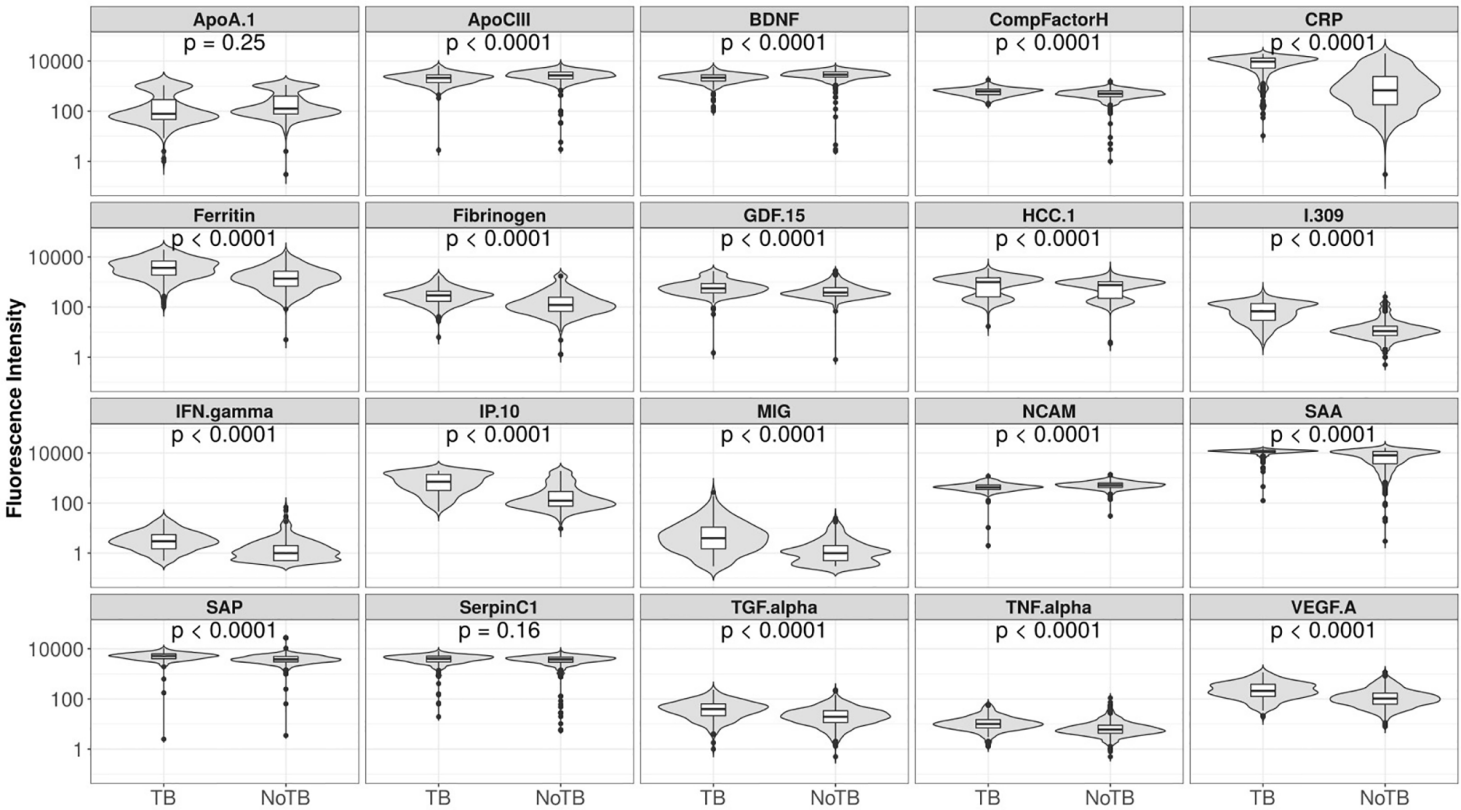
Fluorescent intensities on a log scale of biomarkers detected in the serum samples of participants with tuberculosis (TB) and those with other respiratory disease (ORD), for all 20 markers analyzed. Unadjusted permutation test p-values for the difference between the means of the groups are given for each biomarker (105 permutations).

Using the training data-set, 17 of the 20 serum markers investigated in the study showed significant differences (p<0.0025 corrected for multiple testing) between the TB and ORD cases, irrespective of HIV infection status or country of sample origin. Those that did not were IFN-γ, ApoA-1, and Serpin C1. When we investigated the ability of the markers to diagnose TB disease using ROC curve analysis, the areas under the ROC curve (AUC) were between 0.70 to 0.86 for 12 out of the 20 investigated analytes, namely; ApoCIII, BNDF, I-309, CRP, IP-10, MIG, ferritin, fibrinogen, IFN-γ, SAA, SAP, and TNF-α (**Figures 2** and **3**).

### Performance of Serum Multi-Analyte Models in the Diagnosis of TB Disease

#### Performance of the Optimal Bio-Signature

In order to identify the best performing marker combination, we generated a model using the sparseLDA algorithm with no constraint on marker number. This identified a two-marker, optimal bio-signature. The selected markers were: I-309 and CRP. We constructed a sparseLDA model from these markers, including interactions between terms, which yielded a sensitivity of 93% and a specificity of 68% on the test set. The area under the ROC curve (AUC) for this model was 0·90 (**Table 4**) (26).

**Table 4:** Performance of the predictive models.

Signature	N	AUC (95% CI)	Sensitivity (95% CI)	Specificity (95% CI)
**CRP**	401	0·85 (0·81-0·88)	0·85 (0·81-0·88)	0·74 (0·54-0·84)
** HIV pos**	85	0·79 (0·70-0·87)	0·83 (0·56-0·93)	0·72 (0·39-0·83)
** HIV neg**	316	0·86 (0·81-0·90)	0·86 (0·79-0·92)	0·70 (0·54-0·85)
**6-marker**	350	0·85 (0·80-0·90)	0·89 (0·81-0·96)	0·60 (0·34-0·82)
** HIV pos**	41	0·80 (0·65-0·92)	0·80 (0·65-0·95)	0·57 (0·14-0·95)
** HIV neg**	309	0·85 (0·79-0·91)	0·88 (0·80-0·96)	0·61 (0·29-0·85)
**Optimal**	401	0·90 (0·86-0·94)	0·93 (0·87-0·97)	0·68 (0·36-0·82)
** HIV pos**	85	0·89 (0·80-0·95)	0·87 (0·73-1·00)	0·64 (0·47-0·89)
** HIV neg**	316	0·90 (0·86-0·94)	0·91 (0·84-0·98)	0·73 (0·40-0·89)
** No Previous TB**	338	0·92 (0·88-0·95)	0·94 (0·89-0·99)	0·69 (0·36-0·87)
** HIV pos**	64	0·92 (0·84-0·97)	0·95 (0·85-1·00)	0·64 (0·50-0·89)
** HIV neg**	274	0·92 (0·87-0·96)	0·93 (0·86-0·99)	0·74 (0·43-0·92)
** Previous TB**	63	0·83 (0·70-0·93)	0·81 (0·67-1·00)	0·60 (0·29-0·93)
** HIV pos**	21	0·84 (0·62-0·97)	0·70 (0·40-1·00)	0·73 (0·27-1·00)
** HIV neg**	42	0·79 (0·60-0·95)	0·82 (0·45-1·00)	0·68 (0·13-0·97)
**Optimal Excluding CRP**	401	0·89 (0·85-0·94)	0·92 (0·86-0·97)	0·70 (0·47-0·84)
** HIV pos**	85	0·88 (0·79-0·94)	0·93 (0·77-1·00)	0·56 (0·40-0·84)
** HIV neg**	316	0·89 (0·84-0·93)	0·89 (0·81-0·96)	0·77 (0·52-0·92)

Signatures: Six-marker (ApoA-1, CFH, CRP, IFN-γ, IP-10, SAA), optimal (I-306 and CRP), optimal excluding CRP (I-309, NCAM and SAA). N=test-set samples.

**Figure 4:**
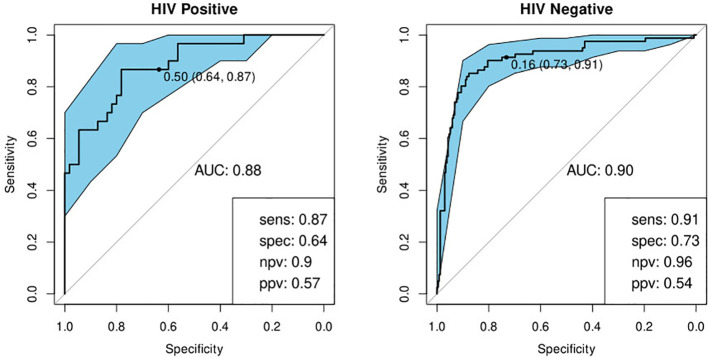
Receiver-operator characteristic (ROC) curve for the optimal bio-signature (CRP and I-309) for HIV-positive (N=85) and HIV-negative (N=316) individuals. The optimal sensitivity and specificity are shown on the bottom right corner together with the negative and positive predictive values.

While training the model, we noted that the optimal cut-off for HIV-positive participants was different to that for HIV-negatives. In order to maximize the performance of the model, it was therefore necessary to choose separate cut-off values for these two groups. The model then yielded sensitivities and specificities of 91 and 73% for HIV-negative cases and 87 and 64% for HIV-positive cases respectively (**Table 4**; **Figure 4**). Attempting to improve the model’s performance on HIV-positive cases by weighting their contribution to the model by the inverse of their proportion during training did not produce any noticeable effect.

#### Performance of the Best Bio-Signature Without CRP

To determine the degree of dependence on CRP, we repeated the optimal model construction, excluding CRP from the available set of markers. In this scenario, the algorithm selected I-309, NCAM and SAA. The performance of this signature was marginally inferior to, that of the optimal signature in both HIV-negative and HIV-positive individuals (**Table 4**, **Figure S3**). Weighting the data to increase the contribution of HIV-positive cases did not significantly improve the performance of the model on this group.

#### Performance of the Previously Identified Seven-Marker Bio-Signature

**Figure 5:**
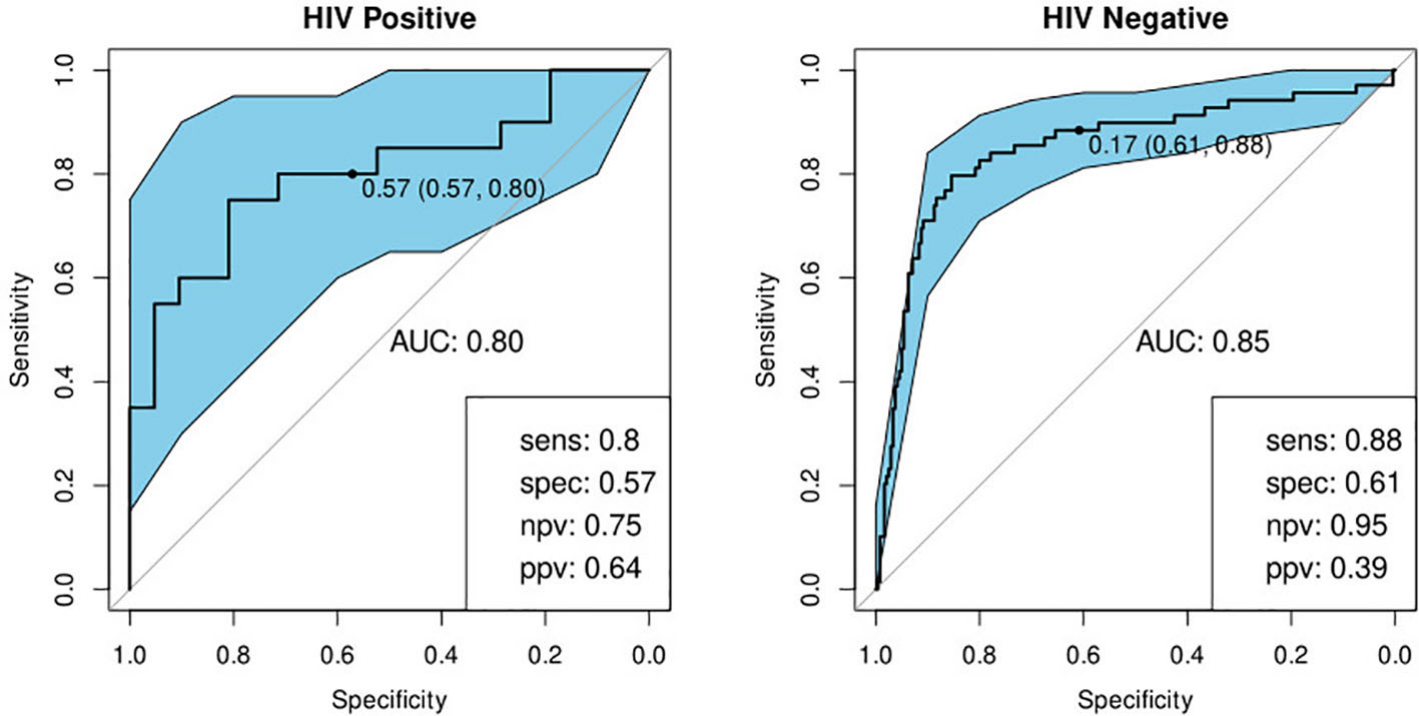
Receiver-operator characteristic (ROC) curve for the modified seven marker bio-signature originating from Chegou and colleagues, excluding transthyretin, for HIV-positive (n=41) and HIV-negative (n=309) individuals (20). The optimal sensitivity and specificity bottom right together with the negative and positive predictive values.

We previously identified a seven-marker bio-signature comprising: ApoA-1, CFH, CRP, IFN-γ, IP-10, SAA, and transthyretin (20). This had a sensitivity and specificity of 93.8 and 73.3% respectively when the model was applied to a test set. The data for that study was generated from an early-recruitment subset of the samples used in the current study, from five of the seven field-sites. We investigated the diagnostic potential of the seven-marker serum bio-signature, with transthyretin removed, as antibodies for this marker were no longer available for Luminex^®^. To do this, we used a 60% training set which was a super-set of samples from the participants common to this and the original study. Model performance was evaluated on a test set comprising the remainder of the samples in the current study. These had not been part of the original study. This signature did not perform well. The sensitivity and specificity in HIV-negative individuals was 88 and 61% respectively and in HIV-positive individuals, 80 and 57% (**Table 4**; **Figure 5**).

#### Performance of CRP

**Figure 6:**
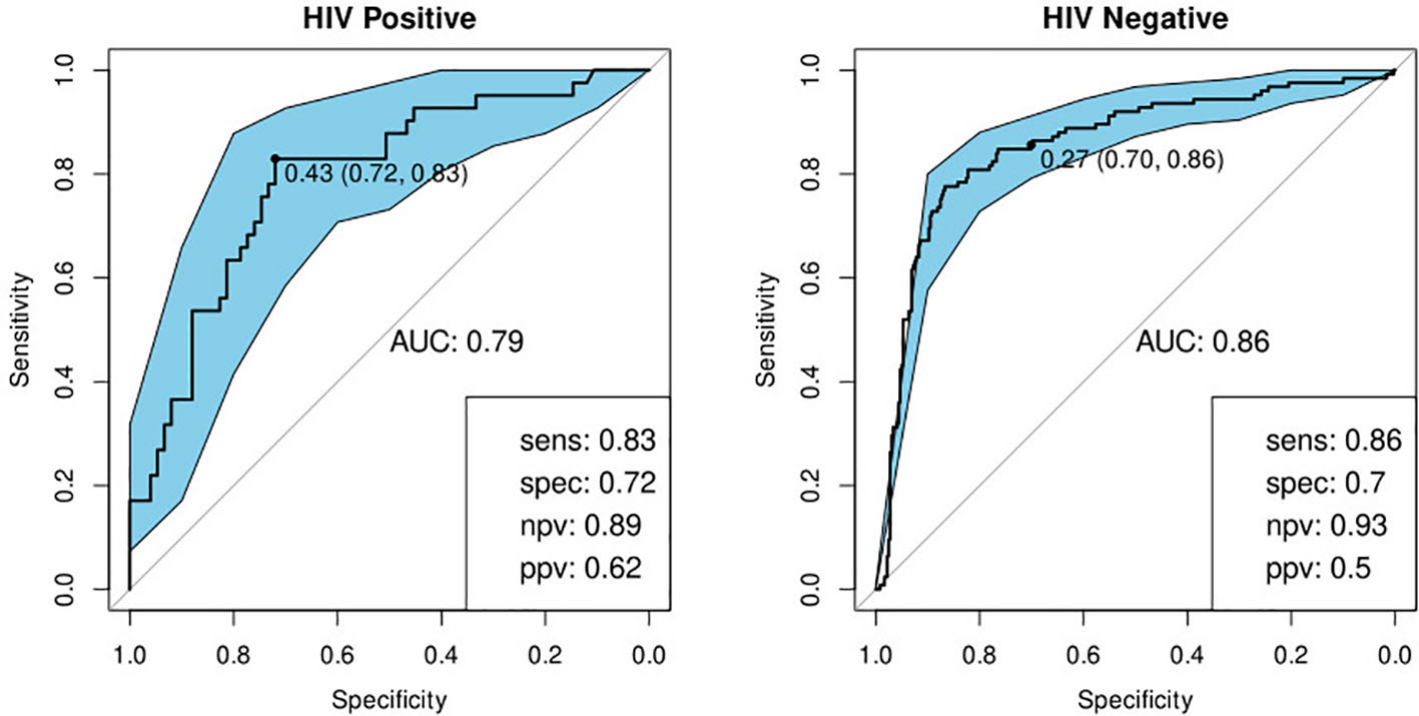
Receiver-operator characteristic (ROC) curve for CRP only for HIV-positive (N=85) and HIV-negative (N=316) individuals. The optimal sensitivity and specificity are shown on the bottom right corner together with the negative and positive predictive values.

We built a model to validate the previously identified single diagnostic marker, CRP (22). We found a sensitivity of 89% and specificity of 75% in HIV-negative participants and 90 and 67% respectively when tested against HIV-positive participants (**Table 4**; **Figure 6**).

#### Performance with Respect to History of Previous TB

**Figure 7:**
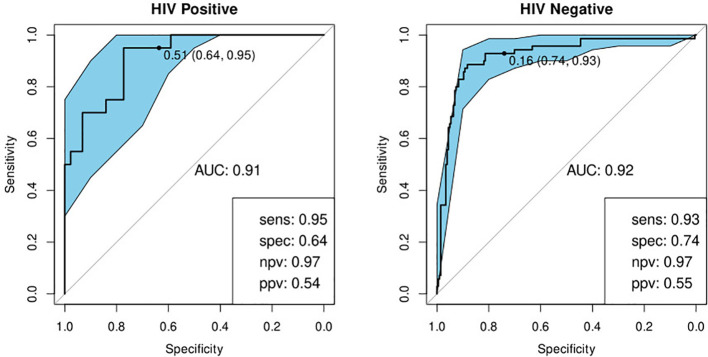
Receiver-operator characteristic (ROC) curves of the performance of the optimal bio-signature (CRP and I-309) for HIV-positive (n=64) and HIV-negative (n=274) individuals with no previous history of TB. The optimal sensitivity and specificity are shown on the bottom right corner of each ROC curve together with the negative and positive predictive values.

**Figure 8:**
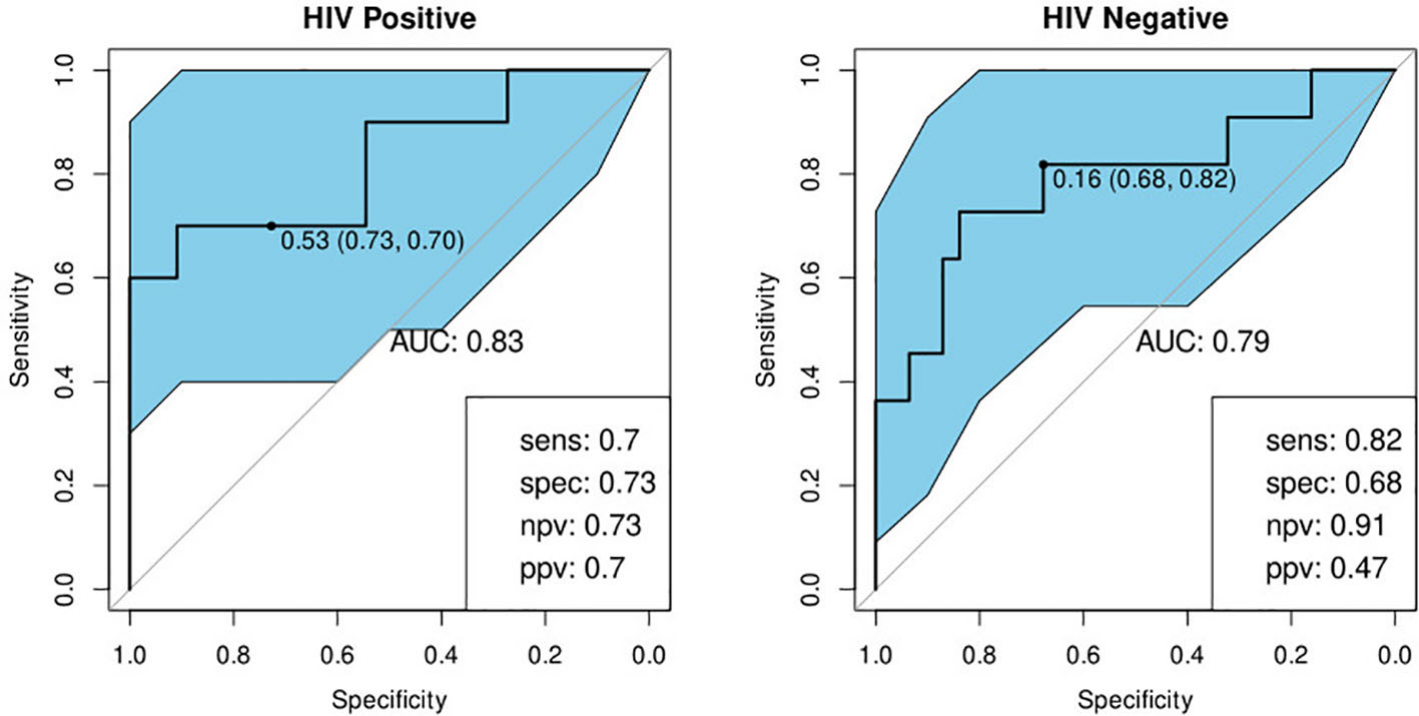
Receiver-operator characteristic (ROC) curves of the performance of the optimal bio-signature (CRP and I-309) for HIV-positive (n=21) and HIV-negative (n=42) individuals with a history of previous TB. The optimal sensitivity and specificity are shown on the bottom right corner of each ROC curve together with the negative and positive predictive values.

We investigated the performance of the optimal model separately on participants with a history of previous TB and those without. We found that the bio-signature performed well for participants with no history of previous TB. For HIV-positive participants, we achieved a sensitivity of 95% and a specificity of 64% and for HIV-negatives, a sensitivity of 93% and specificity of 76% (**Table 4**; **Figure 7**). For participants with a previous episode of TB, however, the bio-signature was far less informative. In this case, for HIV-positive participants, we measured a sensitivity of 70% and a specificity of 73%, while for HIV-negatives, the values were 82 and 68% respectively (**Table 4**; **Figure 8**). The optimal bio-signature therefore met the requirements of the WHO TPP for a triage test in patients without a history of previous TB, although the specificity for HIV-positive patients was slightly lower than desired (26).

### Comparison of Model Performance

In comparing the AUCs of the ROC curves of the various models, we found that the optimal signature significantly out-performed CRP on its own (p=0.032, stratified bootstrap). This was also the case when we compared the sensitivities of these two models at a specificity of 0.7 (p=0.032, stratified bootstrap). There were no significant pairwise differences between any of the other models.

## Discussion

The development of a better diagnostic tool for TB is an important goal in the fight against this disease. To this end, we investigated the ability of 20 previously identified host serum protein biomarkers to diagnose TB disease in individuals presenting with symptoms suggestive of TB disease at peripheral healthcare clinics in six African countries. Twelve of the 20 investigated biomarkers showed promise in this study for diagnosing TB disease as their ROC curves when distinguishing between the TB and ORD groups had AUCs greater than 0.7.

Although many biomarkers obtained from various body-fluids show promise individually for diagnosing TB, single markers tend to be less robust than combination bio-signatures due to their non-specificity and tendency to be affected by other factors such as HIV-infection (20). This was the case when we attempted to validate the previously identified biomarker, CRP, which was less effective at diagnosing TB than the optimal two-marker model.

We identified an optimal diagnostic bio-signature, irrespective of African country of origin, comprising two markers: the chemoattractant, I.309 and CRP. This signature meets the WHO minimum requirements for a triage test, which is a sensitivity of 90% and specificity of 70%, in HIV-negative patients and in HIV-positive patients with no previous history of TB (26). Given the frequent co-morbidity of TB and HIV infection and the difficulties this creates for diagnosing TB, it is encouraging that our diagnostic bio-signature is successful regardless of HIV infection status, albeit with wider confidence intervals in the case of HIV positive participants. It should be noted, however, that I.309 is present in blood at far lower concentrations than CRP which poses a technological challenge for measurement in a point-of-care device.

The poor performance that we observed for our bio-signature in participants with a history of previous TB has not, as far as we are aware, been previously reported and raises the question as to whether any surrogate biomarker for TB disease can perform well in patients in this category. It is worth noting that this is a limitation shared by the Xpert Ultra^®^ technology (27). The number of patients falling into this group in the test set was small (n=63) which may explain the lack of significant difference in the performance of the model between this and the first-time TB group. However, it is also possible that this group had an inherently greater variability in marker levels leading to the very wide observed confidence intervals. Considering that most candidate markers for active TB are related to inflammation and that previous TB episodes often cause chronic lung damage, in turn leading to chronic inflammation, it is not entirely unexpected that the performance of these markers could be influenced by previous TB episodes. These preliminary findings will need to be confirmed by further studies. Since it has been established that a prior episode of TB tends to increase the risk of a repeat episode, it could be argued that such patients, on presenting with suspected TB, should bypass the triage test and be referred for confirmatory testing (28). It would be interesting to know whether the time elapsed since the previous episode affects the predictive power of the test. However, this will need to be examined in a follow-up investigation as this data was not available in the present study.

Yoon et al. previously reported on a CRP-based point-of-care screening test for TB (89% sensitivity and 72% specificity with a pre-determined threshold of 10mg/L) in antiretroviral therapy-naive, HIV-infected individuals with low CD4-cell counts from Uganda (22). We found CRP to be a common factor in, and indeed, the most important component of all our bio-signatures. When we excluded this marker from use by the modeling algorithm, we observed a slight, but non-significant, decrease in discriminatory power of the resultant model as has been previously reported (29). While raised levels of CRP are by no means specific to TB, this marker does appear to be the strongest differentiator between this and other lung diseases. An advantage of CRP as a biomarker is its applicability, regardless of HIV infection status, due to its lack of dependence on the presence of CD4 cells (29). This feature, however, is not unique to CRP as other biomarkers such as IP-10, SAA, and ferritin are also produced in the context of HIV infection and may, therefore, be suitable substitutes (30, 31). It seems clear, however, that any serum protein-based bio-signature is likely to require the inclusion of CRP, but that additional markers may aid in boosting its performance above the WHO’s TPP threshold.

Ideally, a point-of-care test would be capable of analyzing a raw, unprocessed sample such as finger-prick blood. Given that the current results are based on serum analyzed using a highly sophisticated laboratory instrument, it is likely that any point-of-care test based on the same analytes would demonstrate different characteristics, resulting in a possible loss of performance in translation to the final product. However, the significant degree of correlation between many immunological biomarkers means that it would be quite possible to substitute a marker which did not perform as desired in the end-product device with an alternative. It should also be noted that the performance of our bio-signatures exceeded the minimum criteria by a considerable degree in three of the sites which suggests that there may be some margin for loss of performance without dropping below these values.

Our attempt to validate our previously reported, seven-marker bio-signature was not successful. This failure may partially be explained by the necessary exclusion of transthyretin, which was an important component of the original signature, but was not available on the Luminex^®^ platform. Nevertheless, this result highlights the importance of subjecting promising results to validation on independent datasets.

A major strength of this study is the diversity of the study sites, including participants from East, West, and Southern Africa. We were able to demonstrate that our bio-signatures performed well in sites from all three African regions, although performance in three sites (KPS, UNAM, and EHNRI) was less than optimal. This is particularly pertinent as Africa accounts for 16 of the 30 countries with a high burden of TB and is also subject to resource limitations, making a point-of-care test highly beneficial in this context (1). Site-specific performance of the optimal model is presented in the supplemental data (**Figure S2**).

The under-performance of the bio-signatures in certain sites is a source of some concern. In the case of UNAM, participant numbers were approximately half that of the other sites with the result that there were only 19 participants from this site in the test set. In addition, due to overly rigorous screening at this site, only 22% of participants fell into the ORD group. In addition, UNAM and KPS had much higher proportions of HIV-positive participants than the other sites which may have negatively impacted their results. It should also be noted that these three sites experienced a number of logistical difficulties such as remote location, unreliable power supply, and less experienced staff which may have impacted on the quality of the samples and data.

The pan-African performance of our bio-signature is very encouraging, however, it remains to be seen whether this success translates to other settings where differing conditions or population genetics may have a negative impact. It also remains for the bio-signature identified in this study to be validated using finger-prick blood and technology appropriate to a point-of-care test. Further studies addressing these questions are in progress.

Being blood-based, rather than requiring the production of sputum, our bio-signature may also prove to be useful in children, who typically develop paucibacillary disease, as well as in individuals presenting with extra-pulmonary TB. Performance in these cohorts should be addressed once the signature has been validated in field-tests in adult pulmonary TB patients.

A potential source of error in this study, and others of this nature, stems from the existence of a subgroup of participants whose diagnosis is uncertain. A rule-out test, such as the one proposed here, ought to classify these as TB so that they would be subject to further investigation. Unfortunately, this cannot be verified as these participants were excluded from this study. This is a point which should be addressed in a field-trial of the diagnostic test.

We suggest that the results presented here constitute strong evidence that the development of a TB triage diagnostic test based on a small number of immunological biomarkers is feasible. Such a tool would be of substantial benefit, particularly in under-resourced setting, which tend to have the highest burden of disease, by reducing the number of unnecessary referrals for expensive, confirmatory diagnostics such as GeneXpert^®^. As a result of its rapid turn-around time, it would also have the benefit of reducing the number of TB cases currently lost to follow-up which, in turn, would decrease the infection pressure in the affected communities.

## ScreenTB Consortium

**Stellenbosch University, South Africa**: Gerhard Walzl, Novel N. Chegou, Petri Ahlers, Stephanus T. Malherbe, Gian D van der Spuy, Ilana van Rensburg, Hygon Mutavhatsindi, Portia Manngo, Kim Stanley, Candice I Snyders, Andriette Hiemstra, Shirley McAnda, Marika Flinn, Bronwyn Smith

**Medical Research Council Gambia at LSHTM**: Jayne S Sutherland, Joseph Mendy, Awa Gindeh, Georgetta Mbayo, Ebrima Trawally, Olumuyiwa Owolabi

**Makerere University, Uganda**: Harriet Mayanja-Kizza, Mary Nsereko, Anna-Ritah Namuganga, Saudah Nambiru Kizito

**Armauer Hansen Research Institute, Addis Ababa, Ethiopia**: Adane Mihret, Sosina Ayalew, Rawleigh Howe, Azab Tarekegne, Bamlak Tessema

**University of Namibia, Namibia**: Emmanuel Nepolo, Joseph Sheehama, Gunar Gunther, Azaria Diergaardt, Uapa Pazvakavambwa

London School of Hygiene and Tropical Medicine, United Kingdom: Hazel Dockrell

**Leiden University Medical Centre, Netherlands**: Tom Ottenhoff, Elisa Tjon Kon Fat, Shannon Herdigein, Paul Corstjens, Annemieke Geluk, Anouk van Hooij

## Data Availability Statement

The raw data supporting the conclusions of this article will be made available by the authors, without undue reservation.

## Ethics Statement

The studies involving human participants were reviewed and approved by Health Research Ethics Committee, Stellenbosch University. The patients/participants provided their written informed consent to participate in this study.

## AE-TB Consortium

**Stellenbosch University, South Africa:** Gerhard Walzl, Novel N Chegou, Magdalena Kriel, Gian D van der Spuy, Andre G Loxton, Kim Stanley, Stephanus Malherbe, Belinda Kriel, Leigh A Kotzé, Dolapo O Awoniyi, Elizna Maasdorp

**Medical Research Council Gambia at LSHTM:** Jayne S Sutherland, Olumuyiwa Owolabi, Abdou Sillah, Joseph Mendy, Awa Gindeh, Simon Donkor, Toyin Togun, Martin Ota

**Karonga Prevention Study, Malawi:** Amelia C Crampin, Felanji Simukonda, Alemayehu Amberbir, Femia Chilongo, Rein Houben

**Ethiopian Health and Nutrition Research Institute, Ethiopia:** Desta Kassa, Atsbeha Gebrezgeabher, Getnet Mesfin, Yohannes Belay, Gebremedhin Gebremichael, Yodit Alemayehu.

**University of Namibia, Namibia:** Marieta van der Vyver, Faustina N Amutenya, Josefina N Nelongo, Lidia Monye, Jacob A Sheehama, Scholastica Iipinge

**Makerere University, Uganda:** Harriet Mayanja-Kizza, Ann Ritah Namuganga, Grace Muzanye, Mary Nsereko, Pierre Peters

**Armauer Hansen Research Institute, Ethiopia:** Rawleigh Howe, Adane Mihret, Yonas Bekele, Bamlak Tessema, Lawrence Yamuah

**Leiden University Medical Centre, Netherlands:** Tom HM Ottenhoff, Annemieke Geluk, Kees LMC Franken, Paul LAM Corstjens, Elisa M Tjon Kon Fat, Claudia J de Dood, Jolien J van der Ploeg-van Schip

Statens Serum Institut, Copenhagen, Denmark: Ida Rosenkrands, Claus Aagaard.

Max Planck Institute for Infection Biology, Berlin, Germany: Stefan HE Kaufmann, Maria M. Esterhuyse

London School of Hygiene and Tropical Medicine, London, United Kingdom: Jacqueline M Cliff, Hazel M Dockrell

## Author Contributions

HM and GS co-wrote the first draft of the manuscript. GS analyzed the data and wrote the final draft of the manuscript. HM performed the Luminex analysis. SM, JSS, HM-K, AC, DK, RH, AM, JAS, and EN oversaw local data and sample collection. GW, NC, JSS, AG, HM-K, AC, DK, RH, JAS, EN, GG, HD, and PC were responsible for the study design. SM was responsible for the participant clinical classification. GS, SM, AG, and NC interpreted the results. GS and KS managed the central data collection. HM, SM, JSS, AG, HM-K, GG, HD, PC, GW, and NC critically revised the manuscript. All authors contributed to the article and approved the submitted version.

## Funding

This study is part of the EDCTP2 program supported by the European Union (grant numbers IP_2009_32040-AE-TBC, DRIA2014-311-ScreenTB, SRIA2015-1065-PredictTB). (URL: https://www.edctp.org/) These grants were awarded to GW. The funders had no role in study design, data collection and analysis, decision to publish, or preparation of the manuscript. HM was funded by the South African Medical Research council through its Division of Research Capacity Development under the Internship Scholarship Program.

## Conflict of Interest

The authors declare that the research was conducted in the absence of any commercial or financial relationships that could be construed as a potential conflict of interest.
