# Supplementary material for: Validation and Optimization of Host Immunological Bio-Signatures for a Point-of-Care Test for TB Disease
Source: Front Immunol. 2021 Feb 26;12:607827. doi: 10.3389/fimmu.2021.607827 (PMC7952865; doi:10.3389/fimmu.2021.607827)
Supplement: Supplementary file 1 [file DataSheet_1.pdf]

frontiers

Supplementary Material

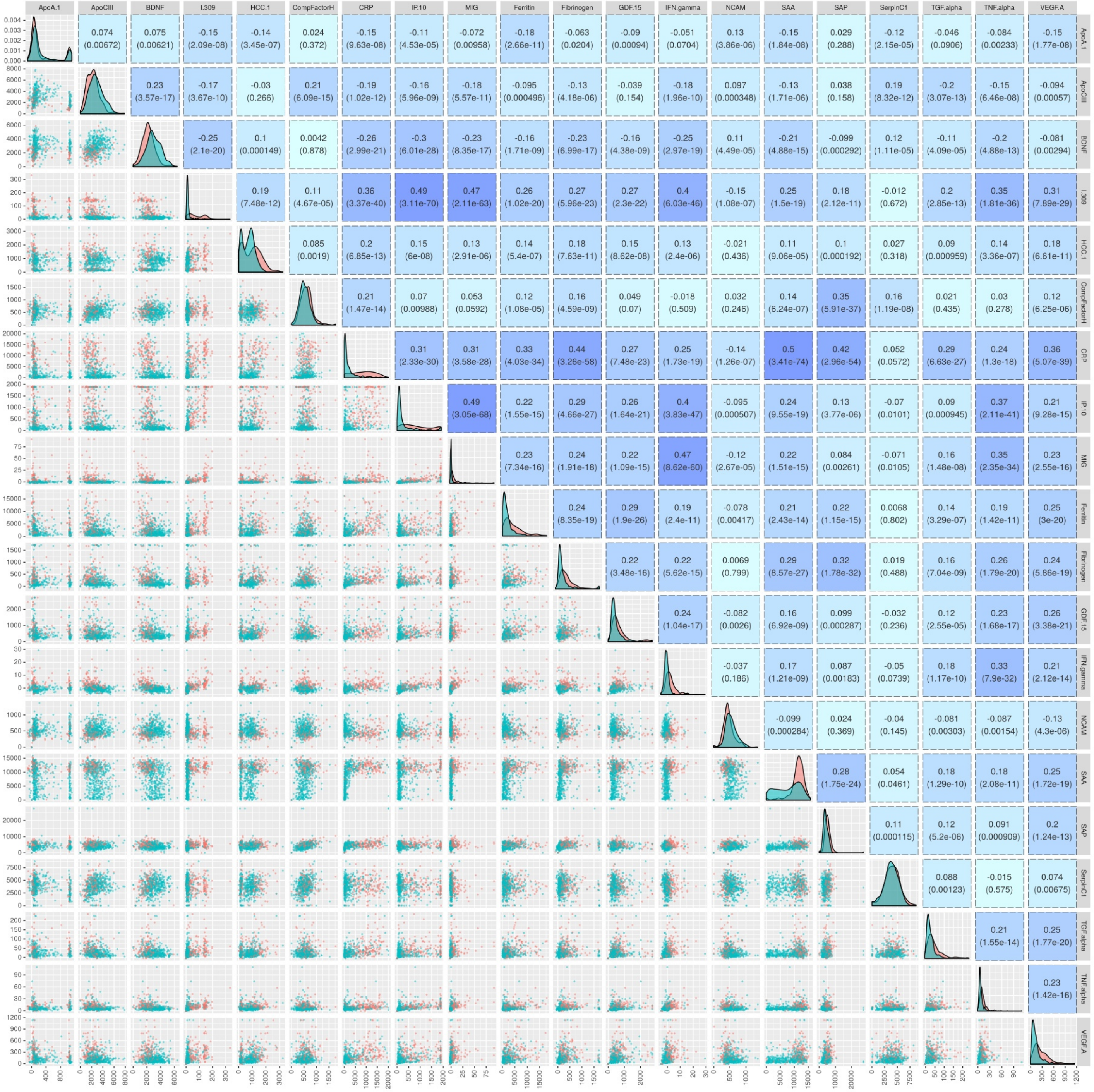

Supplementary Figure 1. Pairwise correlation plot of fluorescence intensity for all markers. Cyan=TB-negative, Pink=TB-positive. The diagonal shows the density distribution for the marker in each column. The upper triangle shows the correlation coefficient with the p-value in parentheses. Darker background colour indicates higher correlation.

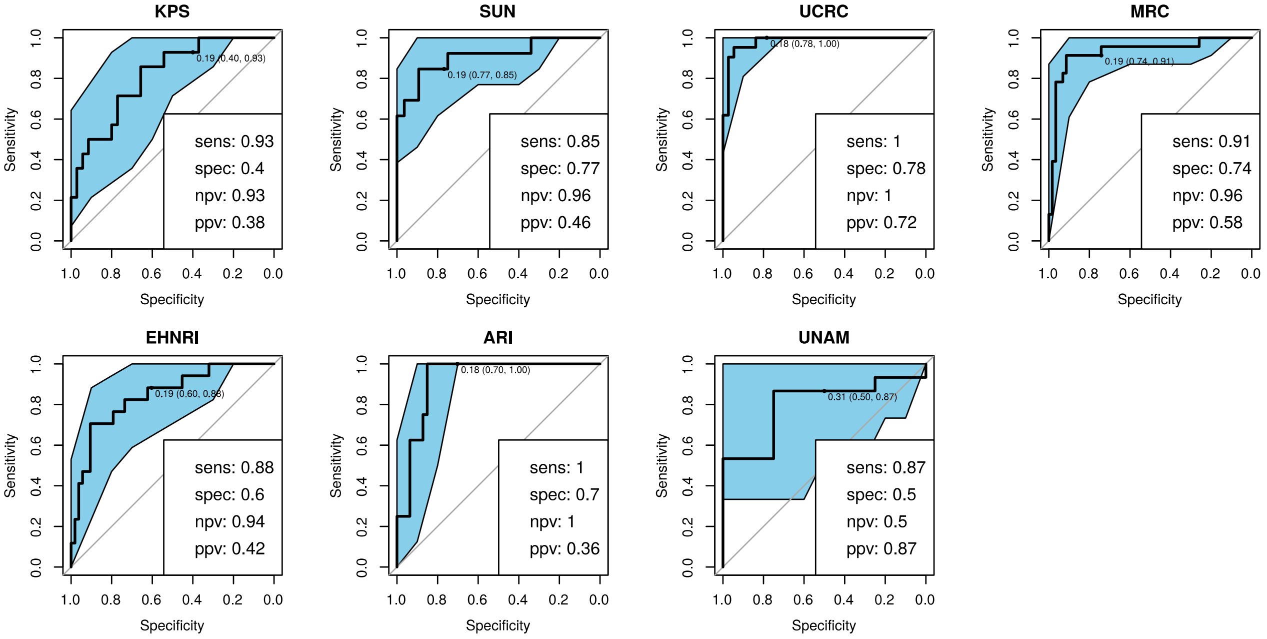

Supplementary Figure 2. ROC curve for the optimal bio-signature (CRP and I-309) for all individuals by study site. The optimal sensitivity and specificity are shown on the bottom right corner together with the negative and positive predictive values.

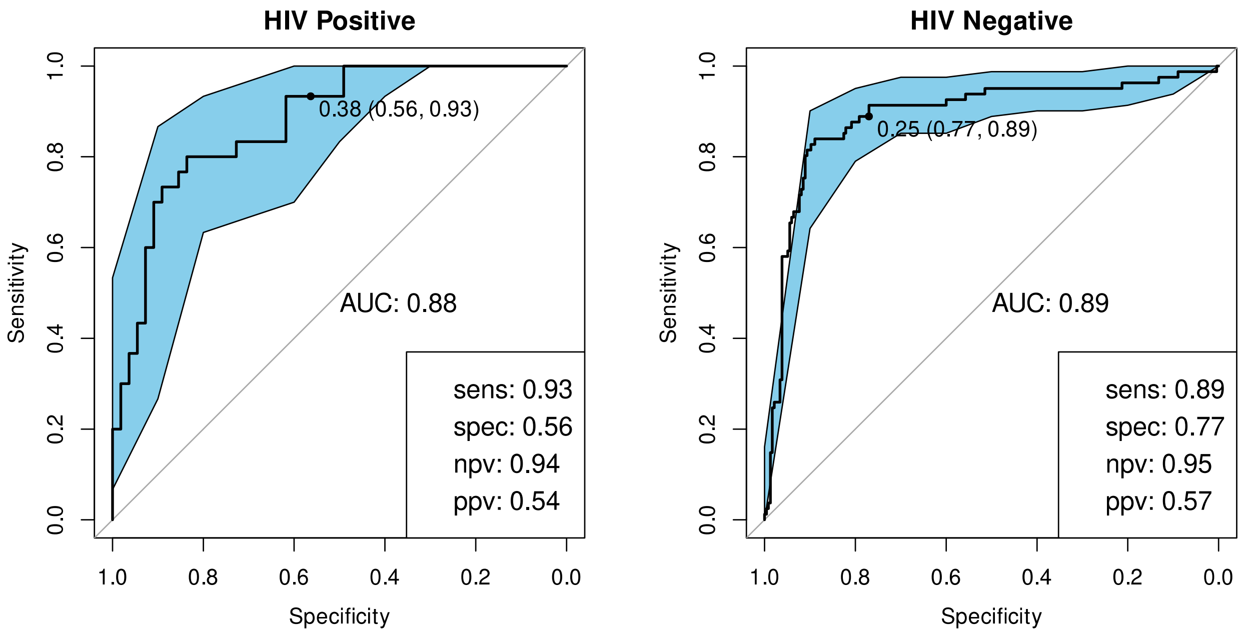

Supplementary Figure 3. ROC curve for the optimal bio-signature excluding CRP for HIV-positive and -negative individuals. The optimal sensitivity and specificity are shown on the bottom right corner together with the negative and positive predictive values.
